# Association of airway CCL28 with type 2 inflammation and secretory IgA in patients with asthma

**DOI:** 10.1371/journal.pone.0361651

**Published:** 2026-10-28

**Authors:** Xiaohan Jiang, Jinmei Zhang, Ke Xu, Xiaobing Yang, Yuanfei Yan, Hao Chai, Jiyong Ma, Ying Shi

**Affiliations:** 1 Nanjing Medical University, Nanjing, China; 2 Department of Respiratory Medicine, Nanjing First Hospital, Nanjing Medical University, Nanjing, China; 3 Department of Cardiology, Nanjing YuHua Hospital, Nanjing, China; 4 Department of Pathology, Nanjing First Hospital, Nanjing Medical University, Nanjing, China; 5 Department of Respiratory Medicine, Nanjing YuHua Hospital, Nanjing, China; 6 Department of Thoracic Surgery, Nanjing First Hospital, Nanjing Medical University, Nanjing, China; Rutgers Biomedical and Health Sciences, UNITED STATES OF AMERICA

## Abstract

**Objective:**

This study aimed to investigate sputum CCL28 expression in patients with acute asthma exacerbations and its association with type 2 airway inflammation and secretory IgA. We further evaluated whether sputum CCL28, alone or in combination with routine clinical indicators, could assist in identifying eosinophilic asthma.

**Methods:**

This study included 75 patients with non-severe acute asthma and 27 healthy controls. Induced sputum samples were collected, and the levels of CCL28, IL-4, IL-5, IL-10, IL-13, and secretory immunoglobulin A (sIgA) were measured in both sputum and serum samples. Sputum cytology was performed to classify airway inflammatory phenotypes as eosinophilic asthma (EA), neutrophilic asthma (NA), mixed granulocytic asthma (MA), and paucigranulocytic asthma (PA). Intergroup differences were analyzed using nonparametric tests, and correlations between variables were assessed using Spearman’s rank correlation analysis. A predictive model for asthma phenotype identification was subsequently developed, and its predictive performance and potential clinical utility were further evaluated.

**Results:**

CCL28 levels in induced sputum were significantly higher in patients with asthma than in healthy controls (*P* < 0.01), while the correlation between sputum and serum CCL28 levels was weak (ρ = 0.29). Sputum levels of IL-4, IL-5, IL-13, and secretory immunoglobulin A (sIgA) were significantly increased, whereas IL-10 levels were decreased (*P* < 0.01). CCL28 levels were significantly higher in the EA group than in the NA and PA groups (*P* < 0.05) and were positively correlated with IL-5, IL-13, sIgA, and sputum eosinophils (r = 0.83, 0.75, 0.66, and 0.55). In the EA prediction model, the combination of CCL28, fractional exhaled nitric oxide (FeNO), and lung function parameters showed the best predictive performance (AUC = 0.79). Incorporation of CCL28 improved model discrimination and reclassification performance (IDI = 0.246, continuous NRI = 0.880, *P* < 0.001) suggesting additional predictive value beyond routine clinical indicators, and decision curve analysis demonstrated a greater net clinical benefit.

**Conclusion:**

Sputum CCL28 was associated with type 2 inflammation and airway mucosal immunity in patients with asthma. An exploratory prediction model incorporating sputum CCL28 showed potential value in assisting the identification of eosinophilic asthma.

## Introduction

Bronchial asthma is a common chronic airway inflammatory disease, and its incidence has continued to rise worldwide in recent years. At present, long-term treatment mainly relies on inhaled glucocorticoids or combined with long-acting inhaled beta2 receptor agonists [1]. However, about 10% of patients find it difficult to control their condition even under high-dose treatment [2], which suggests that the traditional treatment model does not apply to all phenotypes of asthma. One possible explanation is the presence of non-eosinophilic airway inflammation [3]. Based on the proportions of inflammatory cells in induced sputum, asthma can be classified into four inflammatory phenotypes: eosinophilic asthma (EA), neutrophilic asthma (NA), mixed granulocytic asthma, and paucigranulocytic asthma (PA) [4]. In the real world, the types of airway inflammation of asthma are more complex and not immutable. Affected by many factors, smoking, infection, and acute aggravation may cause changes in airway inflammation, leading to different treatment plans, such as antibacterial drugs, systemic hormones. Current research on airway inflammation in asthma has mainly focused on eosinophils, whereas studies investigating biomarkers reflecting airway mucosal function remain limited. Therefore, during acute asthma exacerbations, there remains a need to identify novel biomarkers that reflect both airway inflammatory changes and airway mucosal immune status.

In this study, inflammatory mediators, including CCL28, were measured in induced sputum samples collected from patients with asthma during acute exacerbations, and comparisons were made with corresponding serum levels to better reflect local airway inflammatory status. In addition, sputum cytology was performed to classify inflammatory phenotypes, allowing the identification of biomarker characteristics associated with different asthma phenotypes and supporting precise phenotyping and individualized treatment strategies. Furthermore, machine learning approaches were applied to explore whether sputum biomarkers, particularly CCL28, could assist in asthma phenotype identification, an approach that remains insufficiently investigated in previous studies.

CCL28 (chemokine (C–C motif) ligand 28), also known as mucosa-associated epithelial chemokine (MEC), is a member of the CC chemokine subfamily and is highly expressed in mucosal epithelial tissues, including the respiratory tract, gastrointestinal tract, and mammary glands [5]. CCL28 exerts dual biological functions. Through its receptors CCR3 and CCR10 [6], it mediates the chemotactic recruitment of eosinophils [7], IgA-secreting plasma cells (ASCs) [8], and regulatory T cells (Tregs) [9,10], thereby contributing to inflammatory cell recruitment and immune regulation. In mucosal tissues such as the respiratory tract, CCL28 promotes the production of secretory IgA (sIgA), thereby enhancing mucosal barrier function [8].

Previous studies have shown that both adaptive immunity and innate immunity play important roles in the pathogenesis of asthma. The type 2 response in adaptive immunity drives IgE production and allergic inflammation through Th2 cells and the cytokines they secrete, such as IL-4, IL-5, and IL-13 [11]. IL-10 has a bidirectional regulatory effect [12]. In terms of innate immunity, the airway epithelium serves as a physical barrier and can also secrete IL-33 to activate type 2 innate lymphoid cells (ILC2s), promoting the secretion of inflammatory factors such as IL-5 and IL-13 [13].

Therefore, as an important chemokine secreted by the airway epithelium, CCL28 may be an important hub for connecting adaptive immunity and inherent immunity. Previous studies on CCL28 only detected blood specimens of asthmatic children [14], and this study further tested sputum based on previous studies.

Eosinophilic asthma (EA) represents a clinically important asthma phenotype characterized by type 2 airway inflammation and differential response to anti-inflammatory therapy. However, in clinical practice, commonly used biomarkers such as fractional exhaled nitric oxide (FeNO), serum IgE, or individual cytokines alone show limited sensitivity and specificity for accurate phenotype identification.

Therefore, there remains an unmet clinical need for more robust biomarkers that reflect local airway inflammation. Sputum-derived biomarkers may better represent the airway microenvironment; however, their individual diagnostic performance is often insufficient.

In this context, we hypothesized that integrating sputum CCL28 with routine clinical indicators using a machine learning approach may improve the exploratory identification of EA and help evaluate whether CCL28 provides additional information beyond commonly used clinical biomarkers.

## Methods

### Inclusion and exclusion criteria

This was a single-center cross-sectional study. Asthma patients are diagnosed according to the definition of the Global Initiative for Asthma (GINA) [15]. Participants were recruited at Nanjing First Hospital between 01/09/2025 and 31/01/2026. All asthma patients in the group come from the emergency or outpatient department of the First Hospital in Nanjing, meet the ACT score of <20, are in the acute attack period, and do not use oral glucocorticoids and leukotriene regulators within 14 days before entering the group. The inhaled glucocorticoid and long acting β2 receptor agonist (ICS/LABA) scheme were not replaced, and the drug control conditions were clarified. Exclude patients with severe asthma attacks, chronic obstructive pulmonary disease, bronchiectasis, tuberculosis, lung cancer and other respiratory diseases. The healthy control group matched the age and gender and there was no statistical difference. All subjects have improved the relevant examination, conducted questionnaires, and collected samples. This study was approved by the Ethics Committee of Nanjing First Hospital (KY20250819−02). Written informed consent was obtained from all participants before enrollment.

Sample size was determined by consecutive enrollment during the predefined study period. As this was an exploratory biomarker and predictive modeling study, the sample size was primarily based on clinical feasibility and estimation of effect sizes rather than a formal a priori power calculation. Given the limited number of patients in the eosinophilic asthma subgroup, the predictive analyses were intended to assess the potential value of sputum CCL28 and to generate hypotheses for future validation, rather than to establish a clinically definitive prediction model.

### Collection and processing of sputum samples

Induced sputum was collected as follows: 10 min before the operation, inhale 200 μg of salbutamol to dilate the airway, inhale the atomized 4.5% sodium chloride solution for 10 min, and then instruct the subject to cough out the sputum deep in the airway into the sterile sputum cup. Use a 40μm nylon sieve to remove the impurity fragments in the mixture. The filtered filtrate is centrifuged at 1000 rpm for 8 minutes, and the separated supernatant is stored in a −80℃ refrigerator for subsequent related testing. After the cells in the lower layer are made of the smear, it is fixed in 4% polyformaldehyde for 15 minutes, and the HE dye is dyed. After air drying, the neutral gum is sealed to preserve the smear.

### Measurement of cytokines

Enzyme-linked immunosorbent assay (ELISA) kits were used to measure serum CCL28 levels in patients with asthma and the concentrations of CCL28, IL-4, IL-5, IL-10, IL-13, and secretory immunoglobulin A (sIgA) in sputum supernatants from all participants. The kits were obtained from Jonlnbio (CCL28, sIgA; JL11426, JL10115) and ABclonal (IL-4, IL-5, IL-10, IL-13; RK00003, RK00033, RK00012, RK00034). All assays were performed according to the manufacturers’ instructions. All samples are tested for repeated holes, and the average value is taken for statistical analysis. Standard curve fit R^2^ ≥ 0.999. If the coefficient of variation (CV) between the two holes is > 15% or the detection value exceeds the linear detection range of the reagent, the data is excluded.

### Asthma phenotype classification

Observe the number and proportion of various cells in the sputum smear under a high-magnification microscope and record them. The various cell ratios recorded are classified into four types of inflammatory asthma according to the following criteria: EA: eosinophil percentage≥3%; NA: neutrophils percentage≥61%; MA: neutrophils ≥61% and eosinophil percentage≥3%; PA: neutrophils <61% and eosinophils <3%.

### Statistical analysis

All statistical analyses and data visualizations were performed using R software (version 4.5.1). For continuous variables, normality was assessed prior to analysis. Data with a non-normal distribution are presented as the median and interquartile range (IQR). Comparisons between two groups were performed using the Mann–Whitney U test, whereas comparisons among multiple groups were conducted using the Kruskal–Wallis test. Categorical variables are expressed as counts and percentages (%), and comparisons between groups were performed using the chi-square test or Fisher’s exact test, as appropriate.

For correlation analyses, appropriate methods were selected based on data distribution. Pearson correlation analysis was used for normally distributed continuous variables, whereas Spearman’s rank correlation was applied to non-normally distributed variables. The strength of the correlations was evaluated using the correlation coefficient (r).

To explore whether sputum CCL28 could assist in identifying eosinophilic asthma, a random forest algorithm was applied to construct an exploratory phenotype classification model. Given the relatively limited sample size, particular attention was paid to overfitting prevention and model stability. The model consisted of 500 decision trees and was evaluated using 10-fold cross-validation. To avoid potential information leakage, all model evaluations were performed within the cross-validation framework.

Model performance was assessed using receiver operating characteristic (ROC) curve analysis, and the area under the curve (AUC) with corresponding 95% confidence intervals was calculated using bootstrap resampling. No additional class balancing procedures were applied due to the limited sample size, and performance was primarily evaluated using cross-validation-based metrics. The optimal cut-off value, sensitivity, and specificity were determined based on the Youden index.

To evaluate the incremental predictive value of the biomarker CCL28 beyond the clinical baseline model, net reclassification improvement (NRI) and integrated discrimination improvement (IDI) were calculated. Decision curve analysis (DCA) was performed to assess the clinical net benefit across a range of threshold probabilities.

It is important to note that, due to the limited sample size, particularly in the eosinophilic asthma subgroup, the predictive model should be considered exploratory and hypothesis-generating, rather than for immediate clinical application.

The primary R packages used in this study included dplyr (data processing), randomForest and caret (model construction), pROC (ROC analysis), rmda (decision curve analysis), ggplot2 (data visualization), and stats (basic statistical analyses). All statistical tests were two-sided, and *P* < 0.05 was considered statistically significant.

## Results

### Comparison of clinical characteristics

A total of 75 patients with asthma were included in the asthma group, and 27 healthy volunteers served as controls. No significant differences in age or sex were observed between the two groups (*P* > 0.05).

Compared with the control group, patients with asthma exhibited significantly higher FeNO levels (*P* < 0.001). Regarding pulmonary function parameters, post-bronchodilator FEV1/FVC and predicted FEV1 (post-bronchodilator FEV1%pred) were significantly lower in patients with asthma (*P* < 0.001) (Table 1).

**Table 1:** Clinical characteristics of patients with asthma and healthy controls.

Variable	Asthma	Normal	*p* value
**Number**	75	27	–
**Age,years**	41.00 (28.00–58.00)	45.00 (28.50–59.00)	0.794
**Gender (female %)**	58.7%	55.6%	0.774
**FeNO, ppb**	45.00 (31.00–63.00)	14.00 (10.50–19.00)	<0.001
**post FEV1/FVC%**	69.30 (62.15–74.60)	86.40 (83.30–88.00)	<0.001
**post FEV1 pred%**	74.20 (68.80–82.65)	89.90 (87.60–93.57)	<0.001
**smoking, %**	21.3	40.7	0.068
**inpatient, %**	42.7	0	–
**intravenous glucocorticoids, %**	46.7	0	
**newly diagnosed, %**	40	0	–

Among the 75 patients with asthma, 25 were classified as EA, 25 as PA, 22 as NA, and 3 as MA. Because only three patients were classified as MA, this group was retained in the tables and figures for descriptive purposes only and was excluded from all inferential comparisons among asthma phenotypes. After excluding the MA group from inferential comparisons, significant differences were observed among the EA, PA, and NA groups in FeNO levels (*P* = 0.017), post-bronchodilator FEV1%pred (*P* = 0.021), and post-bronchodilator FEV1/FVC (*P* < 0.001). No significant differences were observed in age, sex, serum IgE, or peripheral blood cell counts among the EA, PA, and NA groups (Tables 2 and 3).

**Table 2:** Clinical characteristics and inflammatory cell profiles of patients with asthma according to inflammatory phenotype.

Variable	EA	PA	NA	MA (descriptive only)	*p* value (exclude MA)
**Number**	25	25	22	3	–
**Age, years**	36.00 (25.00–48.00)	47.00 (37.00–66.00)	41.00 (25.00–57.50)	45.00 (31.50-59.00)	0.081
**FeNO, ppb**	62.00 (44.00-78.00)	42.00 (29.00-53.00)	39.50 (30.00-45.75)	46.00 (32.50-76.50)	0.017
**post FEV1 pred%**	74.20 (65.10-77.80)	78.60 (73.40-84.70)	69.85 (60.35-74.90)	81.80 (77.10-84.90)	0.021
**post FEV1 / FVC%**	69.40 (66.30-73.20)	72.70 (70.90-79.80)	55.55 (49.95-63.35)	71.20 (69.70-87.60)	<0.001
**IgE, KU/L**	322.00 (86.00-495.00)	271.67 (73.16-520.56)	339.22 (216.25-724.00)	724.00 (401.60-2115.00)	0.631
**WBC,10** ^**9**^ **/ml**	8.88 (7.88-11.07)	8.32 (6.74-11.07)	9.29 (6.99-10.50)	9.16 (8.44-9.72)	0.729
**NEU,10** ^**9**^ **/ml**	5.53 (4.97-7.90)	4.89 (3.64-6.57)	5.58 (4.62-7.56)	6.32 (5.52-7.67)	0.371
**NEU%**	64.40 (58.30-75.70)	61.10 (51.80-64.00)	65.75 (55.05-73.35)	69.00 (65.00-78.35)	0.097
**EOS,10** ^**9**^ **/ml**	0.41 (0.08-0.67)	0.55 (0.12-0.86)	0.19 (0.03-0.43)	0.12 (0.07-0.20)	0.131
**EOS%**	3.90 (0.90-7.00)	6.00 (2.20-10.00)	2.35 (0.93-6.12)	1.30 (0.70-2.45)	0.098
**Sputum NEU%**	9.80 (5.30-21.10)	1.80 (1.40-3.30)	68.95 (67.05-70.62)	68.80 (68.10-70.75)	<0.001
**Sputum EOS%**	8.40 (6.40-12.20)	0.10 (0.00-0.70)	0.85 (0.70-1.10)	7.60 (5.75-10.35)	<0.01
**Sputum MA%**	70.30 (65.50-76.40)	89.20 (87.30-93.60)	24.85 (22.15-27.45)	11.70 (10.80-11.90)	<0.01
**Sputum LY%**	4.50 (1.60-7.70)	6.00 (3.70-10.50)	6.60 (2.85-8.35)	11.70 (9.95-12.30)	0.182
**Gender (Male %)**	48%	40%	27.3%	0%	0.344

**Table 3:** Comparison of clinical indicators among patients with different types of asthma.

Variable	EA	PA	NA	MA
**smoking, n**	7	4	5	0
**inpatient, n**	8	12	10	2
**glucocorticoids, n**	8	13	12	2
**newly diagnosed, n**	8	8	12	2

### Comparison of cytokines and sIgA between the asthma and control groups

Compared with healthy controls, sputum CCL28 levels were significantly higher in patients with asthma (*P* < 0.01). The distribution of sputum CCL28 levels was relatively concentrated in the control group, whereas greater variability was observed in the asthma group (Fig 1). Similarly, sputum levels of IL-4, IL-5, IL-13, and sIgA were significantly higher in the asthma group than in controls (IL-4: *P* < 0.05; IL-5: *P* < 0.01; IL-13: *P* < 0.01; sIgA: *P* < 0.01). In contrast, sputum IL-10 levels were significantly lower in the asthma group (*P* < 0.01) (Fig 2).

**Fig 1:**
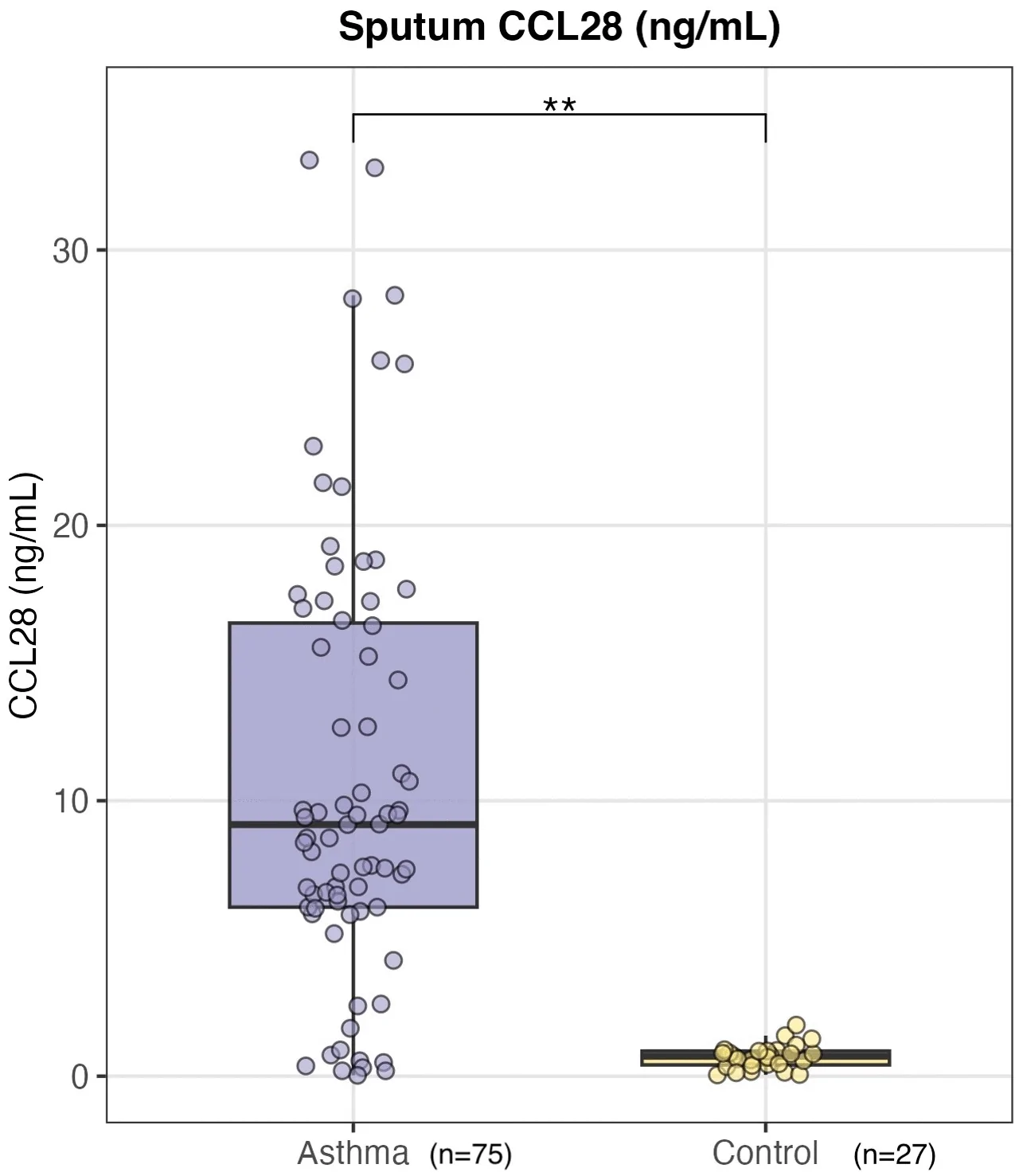
Sputum CCL28 levels in patients with asthma and healthy controls. Sputum CCL28 levels were compared between patients with asthma (n = 75) and healthy controls (n = 27). Data are shown as box-and-whisker plots with individual data points. The horizontal line within each box represents the median, and the box indicates the interquartile range. **P < 0.01.

**Fig 2:**
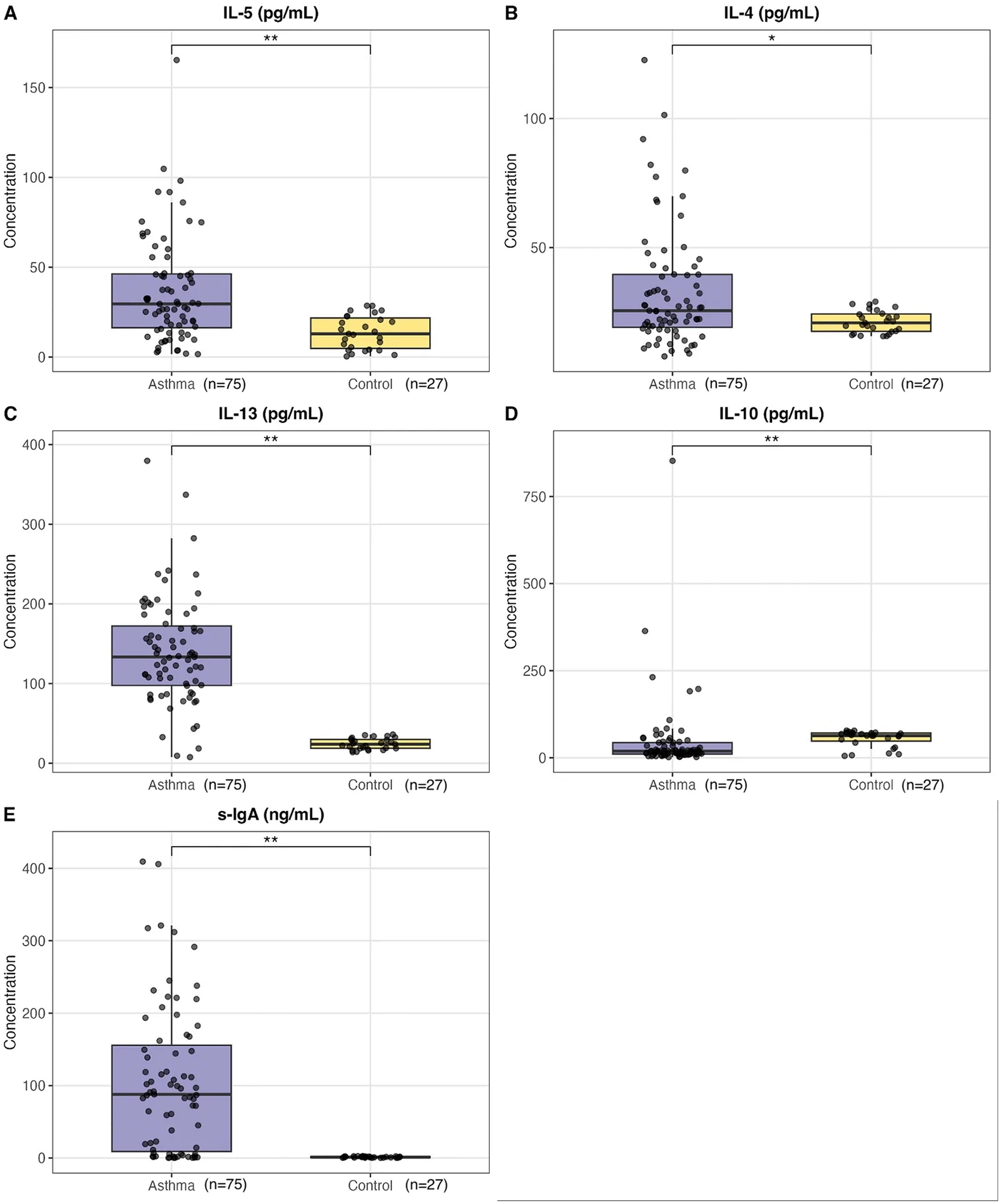
Sputum cytokine and sIgA levels in patients with asthma and healthy controls. Sputum levels of IL-5 (A), IL-4 (B), IL-13 (C), IL-10 (D), and sIgA (E) were compared between patients with asthma (n = 75) and healthy controls (n = 27). IL-4, IL-5, IL-10, and IL-13 are shown in pg/mL, and sIgA is shown in ng/mL. Data are shown as box-and-whisker plots with individual data points. The horizontal line within each box represents the median, and the box indicates the interquartile range. *P < 0.05; **P < 0.01.

### Comparison of cytokines and sIgA among different asthma phenotypes

The correlation between sputum and serum CCL28 levels in patients with asthma was weak (ρ = 0.29) (Fig 3A), and no significant differences in serum CCL28 levels were observed among the phenotypes (Fig 3C). In contrast, sputum CCL28 levels differed significantly among the EA, PA, and NA groups. The highest levels were observed in the EA group, whereas the PA and NA groups showed similar levels. Significant differences were observed between the EA group and both the PA and NA groups (*P* < 0.01) (Fig 3B).

**Fig 3:**
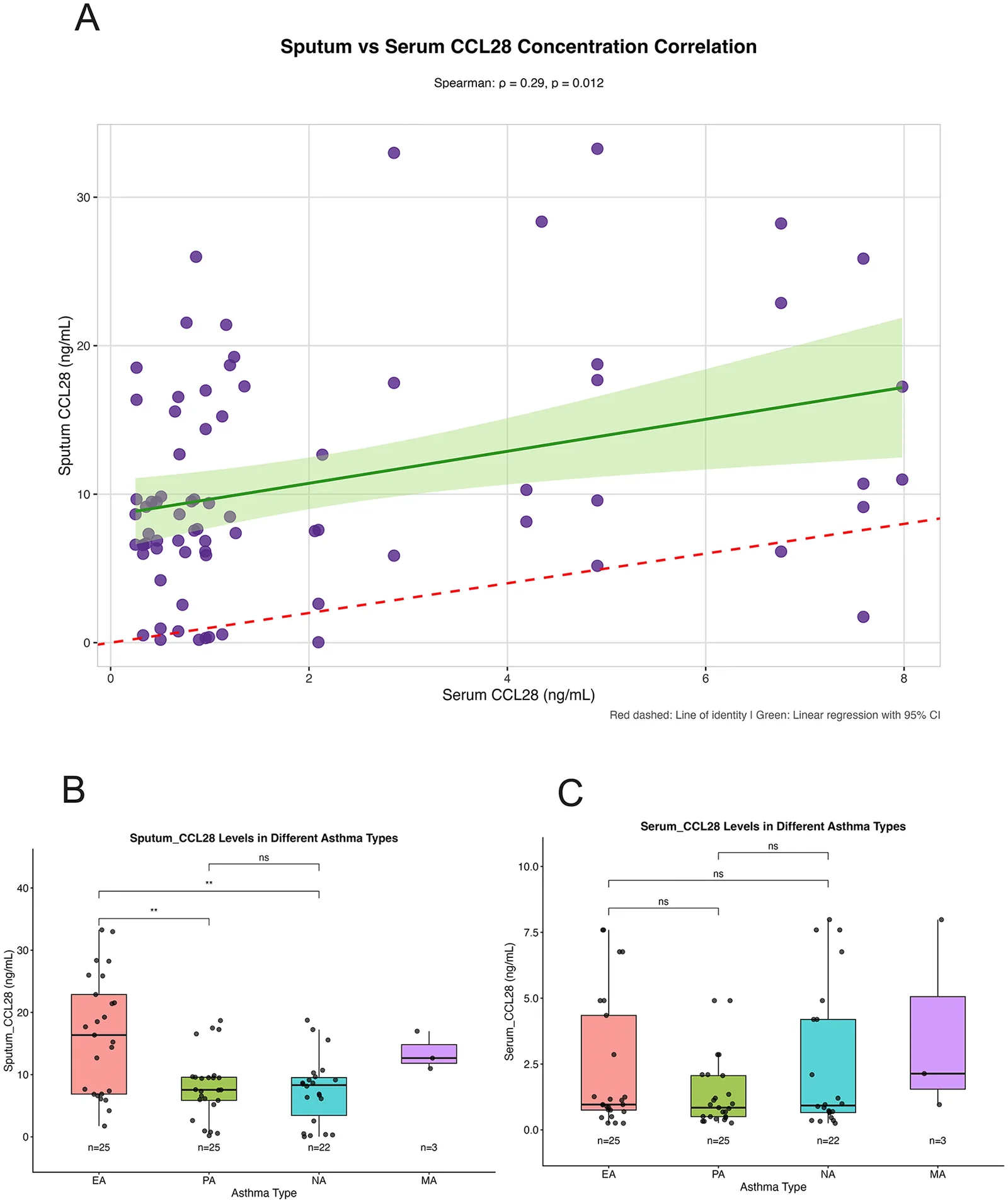
Relationship between sputum and serum CCL28 levels and CCL28 distribution among asthma inflammatory phenotypes. (A) Correlation between sputum and serum CCL28 levels in patients with asthma (n = 75). The correlation was assessed using Spearman’s rank correlation analysis. The green line represents the linear regression fit with 95% confidence interval, and the red dashed line represents the line of identity. (B) Sputum CCL28 levels among asthma inflammatory phenotypes. (C) Serum CCL28 levels among asthma inflammatory phenotypes. Data in panels B and C are shown as box-and-whisker plots with individual data points. The MA group was shown for descriptive purposes only and was excluded from inferential comparisons because of the small sample size (n = 3). Pairwise comparisons were performed among the EA, PA, and NA groups. EA, eosinophilic asthma; PA, paucigranulocytic asthma; NA, neutrophilic asthma; MA, mixed granulocytic asthma. ns, not significant; **P < 0.01.

The distribution of the three Th2 cytokines in sputum differed among asthma phenotypes. IL-4 levels were highest in the EA group and were significantly higher than those in the PA and NA groups (*P* < 0.001). The PA and NA groups showed relatively low levels, with no significant difference between them (Fig 4A). IL-5 levels in the EA group were significantly higher than those in the NA group (*P* < 0.001) and the PA group (*P* < 0.01), whereas no significant difference was observed between the PA and NA groups (Fig 4B). IL-13 levels did not differ significantly among the EA, PA, and NA groups (Fig 4D).

**Fig 4:**
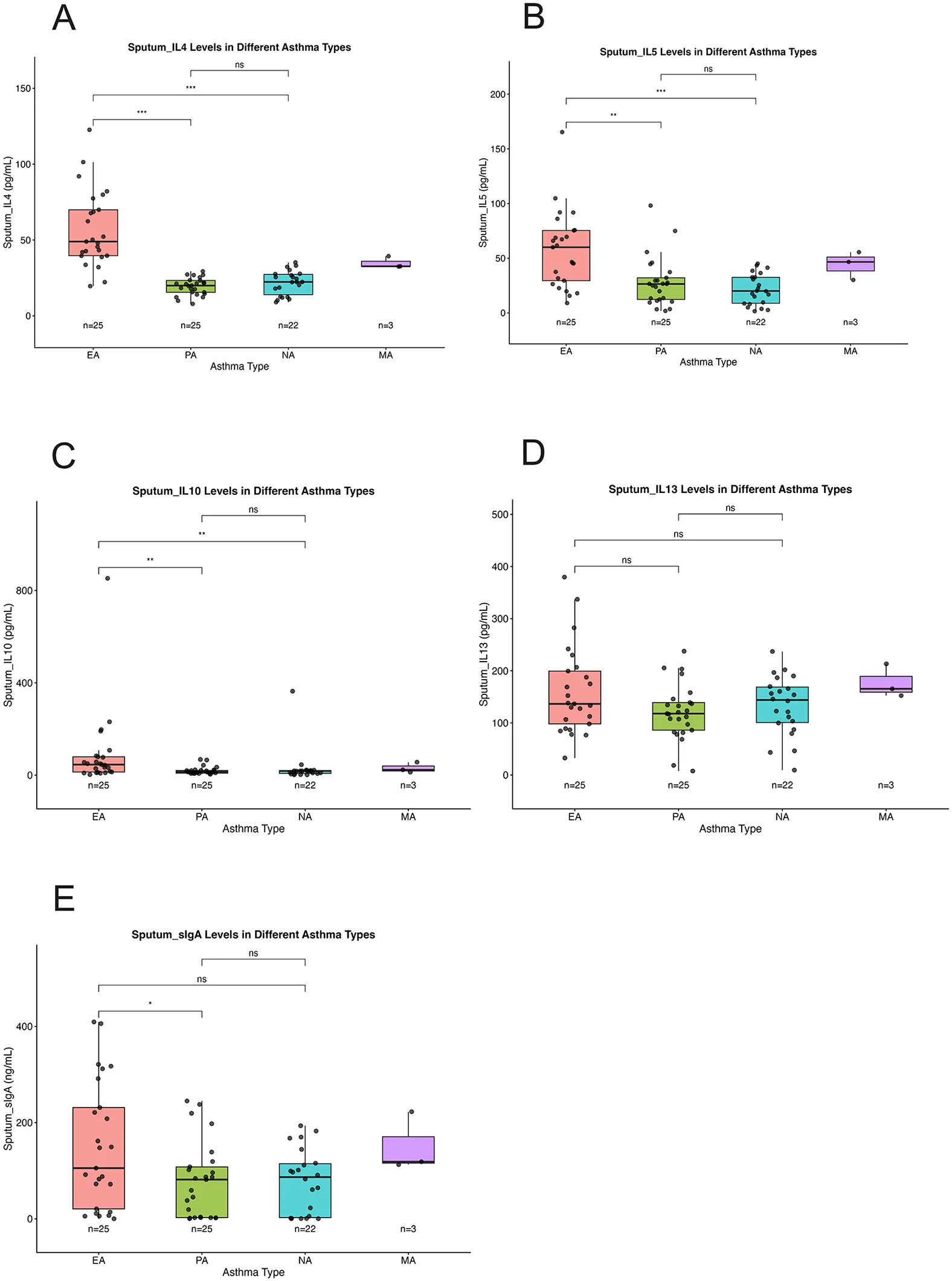
Sputum cytokine and sIgA levels among asthma inflammatory phenotypes. Sputum levels of IL-4 (A), IL-5 (B), IL-10 (C), IL-13 (D), and sIgA (E) were compared among asthma inflammatory phenotypes. IL-4, IL-5, IL-10, and IL-13 are shown in pg/mL, and sIgA is shown in ng/mL. Data are shown as box-and-whisker plots with individual data points. The horizontal line within each box represents the median, and the box indicates the interquartile range. Because only three patients were classified as mixed granulocytic asthma (MA), the MA group was shown for descriptive purposes only and was excluded from inferential comparisons. Pairwise comparisons were performed among the EA, PA, and NA groups. EA, eosinophilic asthma; PA, paucigranulocytic asthma; NA, neutrophilic asthma; MA, mixed granulocytic asthma; ns, not significant; *P < 0.05; **P < 0.01; ***P < 0.001.

Sputum IL-10 levels also differed among asthma phenotypes. The NA and PA groups showed significantly lower levels than the EA group (*P* < 0.01), sIgA levels in the EA group were higher than those in the PA group (*P* < 0.05) (Fig 4E).

### Correlation analysis of various indicators

The results of the correlation analysis are presented as a heatmap (Fig 5). Sputum CCL28 showed strong positive correlations with IL-5, IL-13, and sIgA (r = 0.83, 0.75, and 0.66, respectively). In addition, sputum CCL28 was moderately correlated with the percentage of sputum eosinophils (r = 0.55). A positive correlation was also observed between IL-5 and IL-13 levels (r = 0.64).

**Fig 5:**
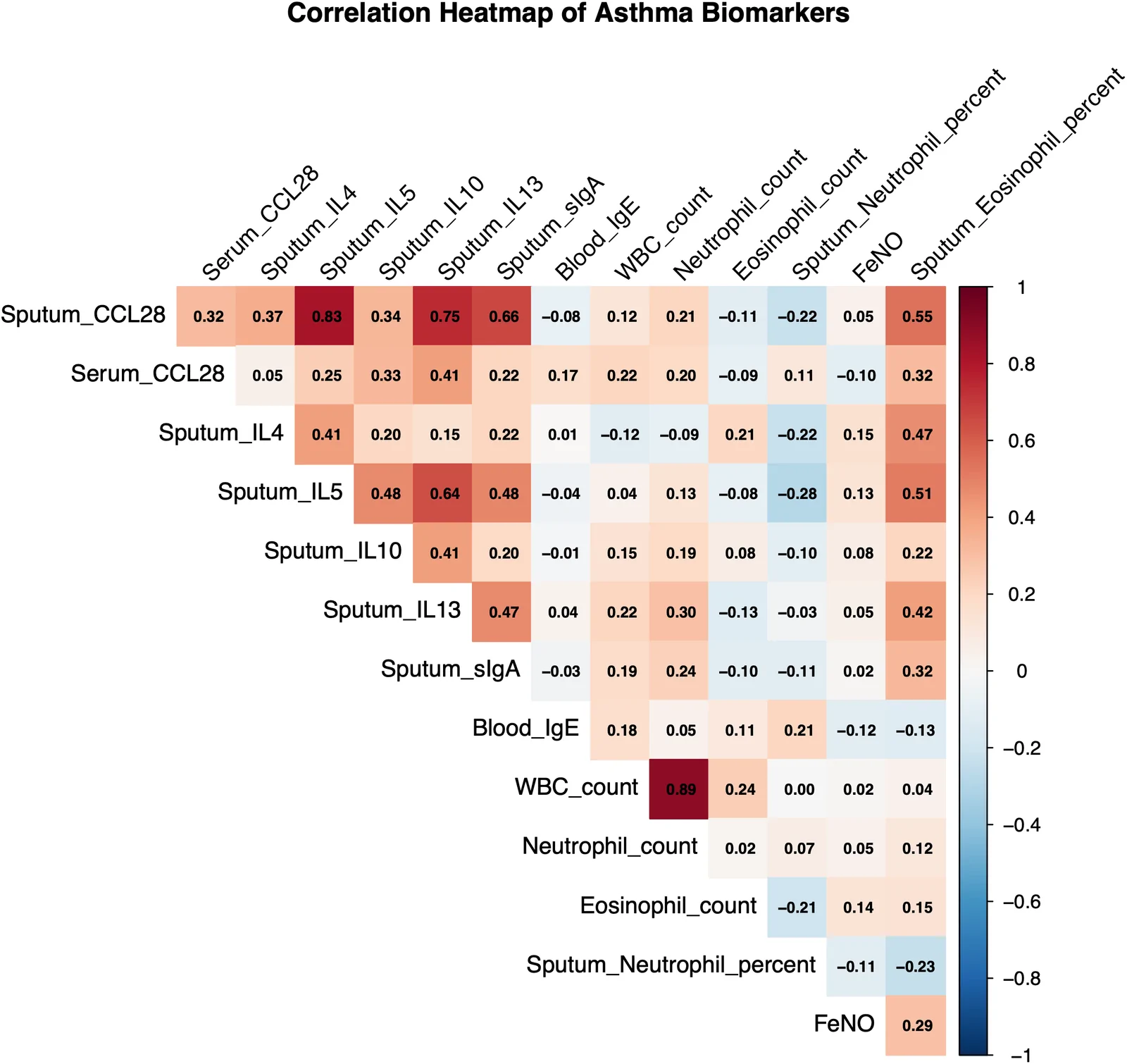
Correlation heatmap of asthma-related biomarkers and clinical indicators. The heatmap shows pairwise correlations among sputum CCL28, serum CCL28, sputum cytokines, sIgA, serum IgE, peripheral blood cell counts, FeNO, and sputum inflammatory cell percentages in patients with asthma. Correlations were assessed using Spearman’s rank correlation analysis. The values shown in each square represent correlation coefficients. Red indicates positive correlations, blue indicates negative correlations, and color intensity reflects the strength of the correlation.

### Construction of a predictive model for identifying the EA phenotype of asthma

ROC analyses were performed to evaluate the ability of sputum CCL28 and related biomarker combinations to identify eosinophilic asthma (EA). As a single biomarker, sputum CCL28 showed moderate discriminative performance, with an AUC of 0.731 (95% CI: 0.597–0.865). The optimal cutoff value was 12.67 ng/mL, corresponding to a sensitivity of 64% and a specificity of 84%.

Among the individual indicators, sputum IL-5 showed the highest AUC (0.786, 95% CI: 0.670–0.901), followed by FeNO (AUC = 0.699, 95% CI: 0.576–0.822). Sputum IL-13, serum IgE, and lung function parameters alone showed relatively limited discriminative ability.

Several combined models were further assessed. The model incorporating CCL28, FeNO, and lung function parameters showed the best overall performance, with an AUC of 0.790 (95% CI: 0.675–0.904), a sensitivity of 76%, and a specificity of 78%. The IL-5 + CCL28 model had an AUC of 0.700 (95% CI: 0.569–0.830), with a sensitivity of 36% and a specificity of 98%. The IL-5 + FeNO model had an AUC of 0.724 (95% CI: 0.604–0.845), with a sensitivity of 44% and a specificity of 90%. The three-marker model combining CCL28, IL-5, and FeNO reached an AUC of 0.760 (95% CI: 0.641–0.879), with a sensitivity of 60% and a specificity of 84% (Table 4, Fig 6).

**Table 4:** Diagnostic performance of individual biomarkers and combined models for identifying eosinophilic asthma.

Models	AUC	95% CI	Cut-off point	Sensitivity (%)	Specificity (%)
**CCL28 + FeNO + lung function**	0.790	0.675-0.904	0.32 (prob)	76	78
**CCL28 + IL5 + IL13**	0.756	0.63-0.882	0.675 (prob)	48	98
**IL-5 + IL-13**	0.671	0.535-0.807	0.495 (prob)	52	80
**Sputum CCL28**	0.731	0.597-0.865	12.67	64	84
**Sputum IL-5**	0.786	0.67-0.901	45.15	64	88
**Sputum IL-13**	0.574	0.428-0.719	167.39	40	78
**Serum IgE**	0.543	0.407-0.679	699	84	30
**Lung function**	0.561	0.421-0.702	78.2	76	40
**FeNO**	0.699	0.576-0.822	34.5	92	42
**IL-5 + CCL28**	0.700	0.569-0.83	0.818 (Prob)	36	98
**IL-5 + FeNO**	0.724	0.604-0.845	0.651 (Prob)	44	90
**CCL28 + IL-5 + FeNO**	0.760	0.641-0.879	0.468 (Prob)	60	84

**Fig 6:**
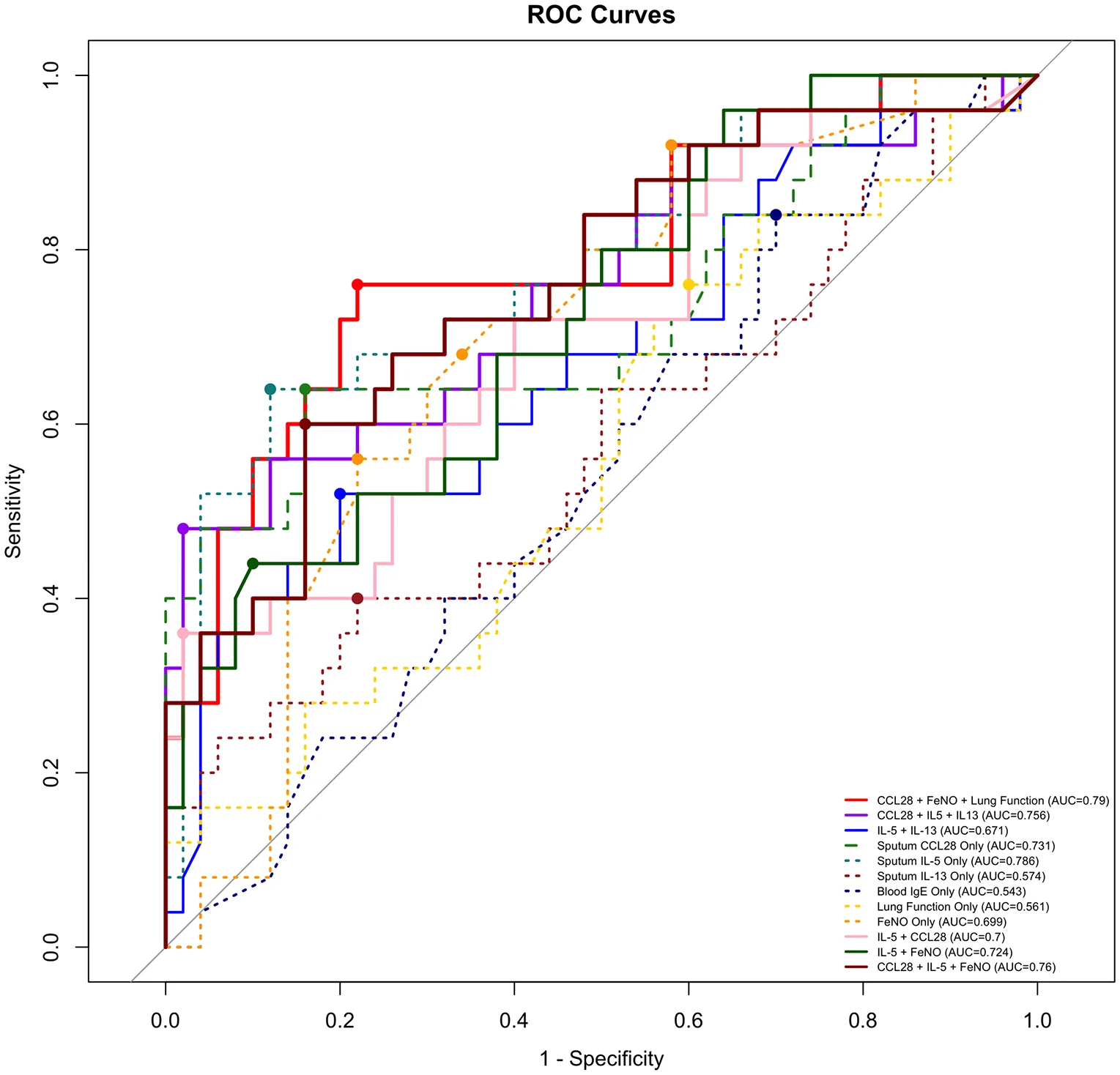
ROC curves for individual biomarkers and combined models for identifying eosinophilic asthma. Receiver operating characteristic (ROC) curves were used to evaluate the discriminative performance of sputum CCL28, sputum IL-5, sputum IL-13, serum IgE, FeNO, lung function parameters, and combined models for identifying eosinophilic asthma. The area under the curve (AUC) values are shown in the legend. The diagonal reference line represents no discriminative ability. ROC, receiver operating characteristic; AUC, area under the curve.

This study further quantified the incremental contribution of sputum CCL28 to the existing clinical evaluation system through the Net Reclassification Index (NRI) and the Integrated Discrimination Improvement Index (IDI). After adding sputum CCL28 to the clinical basic model that only included FeNO and lung function, the discriminative performance of the extended model was significantly improved. Statistical results showed that the Integrated Discrimination Improvement Index (IDI) was 0.246 (*P* < 0.001), and the Continuous Net Reclassification Index (Continuous NRI) was 0.880 (*P* < 0.001). Finally, this study used decision curve analysis to evaluate the net benefit of each model in clinical practice. The analysis results showed that within a wide threshold probability range, the net benefit provided by the extended model including sputum CCL28 was always higher than that of the clinical basic model only including FeNO and lung function (Table 5, Fig 7).

**Table 5:** Incremental predictive value of adding sputum CCL28 to the clinical baseline model.

Indicators	Value	*P* -Value
Integrated Discrimination Improvement (IDI)	0.246	< 0.001
Continuous NRI	0.880	< 0.001
Correlation (r)	0.447	< 0.001

**Fig 7:**
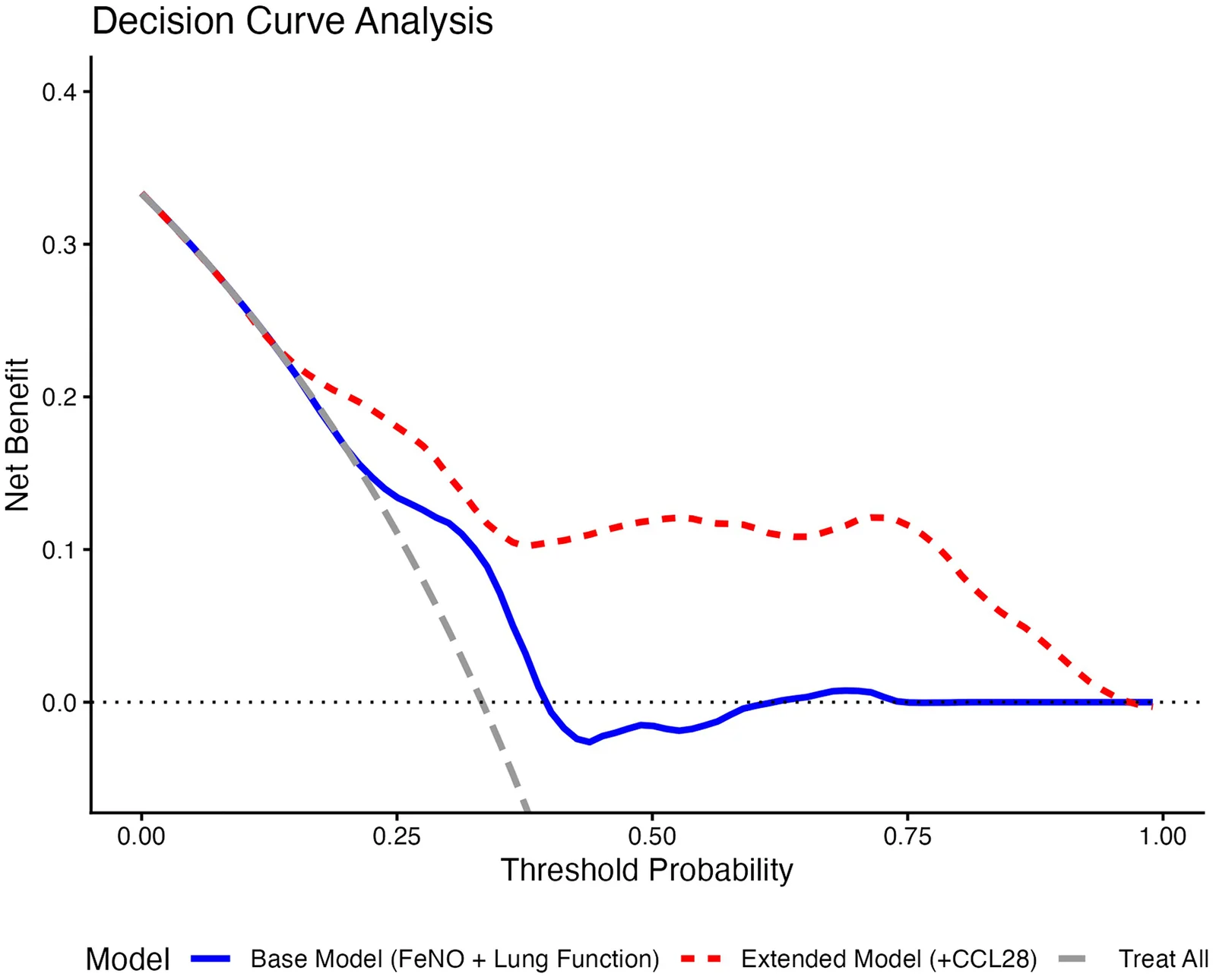
Decision curve analysis of the clinical baseline model and the extended model incorporating sputum CCL28. Decision curve analysis was performed to evaluate the clinical net benefit of the clinical baseline model and the extended model across different threshold probabilities. The clinical baseline model included FeNO and lung function parameters, whereas the extended model additionally incorporated sputum CCL28. The gray dashed line represents the treat-all strategy, and the black dotted horizontal line represents the treat-none strategy. FeNO, fractional exhaled nitric oxide.

## Discussion

This study confirms that CCL28 in the sputum of asthma patients is significantly elevated and is closely related to T2 inflammation, especially in eosinophilic asthma. In addition, the elevation of sputum s-IgA in asthma patients is related to the level of airway CCL28. It is worth noting that we find that sputum rather than serum CCL28 can better reflect the local immune status of the airway. It further highlights the importance of local airway detection.

### Comparison between patients with asthma and healthy controls

The level of CCL28 in the sputum of asthma patients is high but varies greatly from person to person, suggesting that the expression of CCL28 may be affected by the specificity of asthma. Our study shows that there is no obvious correlation between the content of CCL28 in the sputum and serum of asthma patients, which suggests that CCL28, as a chemokine secreted by airway epithelial mucosal cells, is mainly involved in local inflammatory reactions and has limited effect in systemic allergies or inflammatory reactions. Previous studies confirmed that CCL28 was elevated in the serum of patients with irritable bowel syndrome [16], non-small cell lung cancer [17] and atopic dermatitis [14], but most of the relevant studies were limited to peripheral blood levels and lacked local detection and its correlation analysis.

In addition, s-IgA, as an important component of mucosal immunity, plays a role in resisting pathogen invasion and regulating local immune balance. s-IgA is also significantly elevated in sputum in patients with acute asthma attacks, which may reflect the enhancement of local airway mucosal immune activity. This result provides preliminary evidence for the potential role of s-IgA in asthma immune regulation. Studies have found [18] that serum IgA levels are positively correlated with asthma control results, and IgA levels in asthma patients are significantly negatively correlated with the prevalence of moderate and severe asthma. However, the previous research on the relationship between s-IgA and asthma was mostly limited to serum. We have provided new evidence of its role in the immune regulation of asthma through the detection of sputum. CCL28 is closely related to local S-IgA, which can attract plasma cells secreting IgA to migrate to the mucosa and secrete a large amount of IgA into the duct. It is speculated that during the acute aggravation of asthma, the high expression of CCL28 in the airway mucosa is the cause of the increase of S-IgA in sputum.

At the same time, typical T2 cytokines such as IL-4, IL-5 and IL-13 in sputum [19, 20] are also significantly higher than healthy controls, suggesting that asthma patients are dominated by Th2 cell-dominated inflammatory responses. It is worth noting that the level of IL-10 decreased significantly in asthma patients, reflecting that its anti-inflammatory regulatory function may be inhibited, resulting in persistent inflammation. An egg white protein-induced asthma mouse experiment [21] confirmed that IL-10 has a dual role in immune response, which not only inhibits the activation of medullary cells, but also promotes B cell response and epithelial cell proliferation. Another experiment on steroid-insensitive asthma mouse models confirmed that although the administration of IL-10 through the tracheal tract does not affect airway remodeling, it can significantly inhibit the infiltration of eosinophils and neutrophils and the high reactivity of the airway. IL-10 replacement therapy can be used to treat asthma insensitive to steroids [22]. Based on the results of this study, it can be speculated that the decrease in sputum IL-10 level may indicate poor asthma control.

### Comparison among asthma inflammatory phenotypes

The heterogeneity of asthma phenotypes has long been recognized as a major challenge in clinical management. In clinical practice, many patients with asthma are treated using a uniform therapeutic strategy, which may not be appropriate for all phenotypes. For example, neutrophilic asthma is often resistant to corticosteroids and is associated with more severe airway inflammation than eosinophilic asthma [23]. The present study further revealed biological differences among different asthma phenotypes.

In sputum, CCL28 levels were significantly higher in the EA group than in the NA and PA groups. This trend was also observed for IL-4 and IL-5, suggesting that CCL28 is closely associated with eosinophilic airway inflammation and reflects typical type 2 inflammatory characteristics in EA patients. These findings indicate that CCL28 may contribute to the recruitment of eosinophils into the airway via chemokine-mediated pathways. Mechanically, CCL28 can mediate the collection of eosinophils through CCR3 [24], and CCL28 plays a role in regulating the collection of eosinophils to the area around the lung bronchi [25], which is consistent with the results of this study. IL-13 showed no obvious difference between phenotypes but still showed a relatively high level in the EA group, indicating that its role in various types of asthma may be relatively stable or regulated by other factors.

On the contrary, the expression level of IL-10 is the lowest in the NA and PA groups, suggesting that non-T2 asthma patients have impaired their immune regulatory function, resulting in persistent inflammation. Previous studies have shown that IL-10 deficiency can induce excessive inflammatory responses to invading pathogens by promoting the accumulation of neutrophils and Th17 cells in the lungs [26]. In addition, IL-10–producing pulmonary interstitial macrophages have been shown to regulate neutrophil infiltration and exert inhibitory effects on T2 inflammatory responses [27]. These observations are consistent with the findings of the present study.

Correlation analysis further revealed that sputum CCL28 was positively correlated with IL-5, IL-13, sIgA, and the proportion of sputum eosinophils. These findings suggest that sputum CCL28 may serve not only as an independent inflammatory biomarker but also as a central component of the airway inflammatory regulatory network, potentially modulating T2 inflammatory responses through interactions with these mediators.

In the present study, the proportion of patients of NA was relatively high, which may be associated with several factors. Environmental and lifestyle factors, such as air pollution, smoking, and recurrent respiratory infections, may induce neutrophil-dominant inflammatory responses, thereby increasing the prevalence of NA [28]. Neutrophilic asthma is often characterized by poor responsiveness to corticosteroids, persistent inflammatory activity, and an association with greater disease severity. Consequently, this phenotype may be more frequently detected among patients seeking medical care or requiring hospitalization.

In the present study, the number of patients of MA was relatively small, which may be related to the characteristics of the study population and the inclusion criteria. Previous induced sputum cytology studies have demonstrated that EA and NA are the predominant phenotypes, whereas MA and PA occur less frequently, particularly in patients with mild-to-moderate disease [29,30]. Some studies have reported that MA is associated with more severe disease, poor responsiveness to inhaled corticosteroids (ICS), recurrent exacerbations, and airway remodeling, and is therefore more commonly observed in patients with severe or refractory asthma [31,32]. However, in this study, most participants had mild-to-moderate acute exacerbations, and patients requiring intensive care unit (ICU) management were excluded, which may have further reduced the proportion of MA in the cohort. Similar findings have been reported in some studies, showing that MA is associated with more severe lung function impairment and poorer clinical outcomes [33]. Therefore, the relatively low proportion of MA observed in this study may reflect the clinical characteristics of patients with mild-to-moderate acute asthma exacerbations.

### Predictive model

Based on the biomarkers measured in this study, a machine learning approach was introduced and a random forest–based predictive model was constructed to identify patients with EA. In clinical practice, FeNO, an important biomarker of airway inflammation, has been shown to predict eosinophil levels in both sputum and blood [34,35]. IgE, a key mediator produced by B cells in allergic airway diseases, has also been widely studied, and the anti-IgE monoclonal antibody omalizumab has been successfully used in the treatment of allergic asthma [36]. However, these indicators were not retained as core predictors in the final model of the present study. Our analysis showed that several indicators, including FeNO, demonstrated limited predictive performance when used individually. Nevertheless, integrating multiple indicators resulted in improved performance of the multi-biomarker model for predicting EA.

After comparing several combined models, the model incorporating sputum CCL28 with FeNO and pulmonary function parameters showed the best performance for identifying EA, with a sensitivity of 76%, specificity of 78%, and an AUC of 0.79. If the sputum CCL28 is studied alone, the optimal cutoff value determined by the Youden index is 12.67 ng/mL. Under this truncation value, CCL28 shows a high specificity (84%) but its sensitivity is average (64%). Therefore, in practical application, combined with other indicators can improve the accuracy of EA patient identification.

Interestingly, the IL-5-based combination models did not outperform sputum IL-5 alone in this cohort. This may be partly explained by the strong correlation between sputum CCL28 and IL-5, suggesting that these markers may reflect overlapping type 2 inflammatory signals. Therefore, the added value of CCL28 may be more evident when integrated with routine clinical indicators, such as FeNO and lung function, rather than with closely related cytokines alone.

In the present study, the optimal FeNO cutoff value for identifying eosinophilic asthma was 34.5 ppb, which is lower than the widely accepted clinical threshold of 50 ppb. This difference may be attributable to several factors. The commonly used 50 ppb threshold was originally established as a general indicator of type 2 airway inflammation in stable asthma populations, whereas the present cohort consisted of patients experiencing acute exacerbations with more heterogeneous inflammatory characteristics. In addition, the cutoff identified in this study was derived using the Youden index to maximize the discriminatory performance of the model within this specific dataset, rather than to serve as a universal diagnostic standard. Therefore, the FeNO cutoff identified in this study should not be interpreted as a replacement for the established clinical threshold of 50 ppb. Instead, it represents a model-derived operating point for distinguishing eosinophilic asthma within this acute exacerbation cohort, and its clinical relevance requires further validation in larger independent populations.

To further evaluate the incremental value of sputum CCL28, integrated discrimination improvement (IDI) and continuous net reclassification improvement (NRI) were calculated. The addition of CCL28 to the clinical baseline model increased the IDI by 0.246 and the continuous NRI by 0.880, both of which were statistically significant (*P* < 0.001). These findings suggest that sputum CCL28 may provide additional information beyond routine clinical indicators. FeNO mainly reflects type 2 airway inflammation, and pulmonary function parameters reflect the degree of airflow limitation, whereas sputum CCL28 may be more closely related to local mucosal immune activation and chemokine-mediated cell recruitment. Therefore, the combination of these indicators may improve the discriminatory performance for identifying EA, although this finding requires validation in larger independent cohorts.

### Limitations of the study

Although this study demonstrated statistically significant differences in key biomarkers, several limitations should be acknowledged. The study was conducted in a single-center cohort with a relatively small sample size, which may limit the generalizability of the findings. In addition, the number of patients with MA was limited, and therefore this subgroup was only analyzed descriptively without further inferential comparisons.

The predictive model based on random forest was developed using a relatively small dataset and should therefore be interpreted as exploratory. Although internal validation was performed, external validation in independent cohorts is still required to confirm its robustness and clinical applicability. Furthermore, as a cross-sectional study, CCL28 was measured only during acute exacerbations, which does not allow evaluation of longitudinal changes across different disease stages. Future prospective studies with repeated measurements are needed to address this limitation.

## Conclusion

Our findings suggest that the chemokine CCL28, secreted by airway epithelial cells, is significantly upregulated in patients with asthma compared with healthy controls. CCL28 may recruit eosinophils, T cells, and B cells to the lungs, thereby promoting eosinophilic airway infiltration, increased airway sIgA production, and enhanced T2 inflammatory responses, which may contribute to the pathogenesis and progression of asthma. Moreover, CCL28 may serve as a useful biomarker for identifying specific airway inflammatory phenotypes. The exploratory prediction model incorporating sputum CCL28 showed improved discrimination for identifying eosinophilic asthma compared with routine clinical indicators alone. However, given the limited sample size and single-center design, these findings should be interpreted as preliminary and require external validation in larger independent cohorts before clinical application. Overall, this study suggests that sputum CCL28 may be a potential airway biomarker related to type 2 inflammation and mucosal immune activation, providing preliminary evidence for its role in asthma phenotype identification.

## Supporting information

S1 Data Underlying data used in this study. (XLSX)

S1 Code R code used for statistical analyses and predictive modeling. (ZIP)
